# Heavy Grazing Leads to Increased Dominance of Plant and Soil Nematode Communities

**DOI:** 10.1002/ece3.72079

**Published:** 2025-08-31

**Authors:** Rui Dong, Shijie Lv, Changlin Xue, Wentao Wang, Jie Yun, Yanling Wu

**Affiliations:** ^1^ Department of Ecology School of Life Science and Technology, Inner Mongolia Normal University Hohhot Inner Mongolia Autonomous Region China; ^2^ Department of Ecology School of Life Science and Technology, Inner Mongolia Agricultural University Hohhot Inner Mongolia Autonomous Region China

**Keywords:** desert grassland, grazing intensity, nematode community, plant community, the α diversity

## Abstract

Herbivore grazing plays a crucial role in grassland ecosystems, yet its comprehensive impact on plant and soil nematode diversity in desert steppe remains unclear. We assessed the impact of different grazing intensities (CK: no grazing, LG: light grazing, MG: moderate grazing, HG: heavy grazing, EG: extreme heavy grazing) on plant and soil nematode diversity in desert steppe. In the HG treatment, the diversity of plants and nematodes was the lowest and significantly lower than that in the CK treatment. Compared with CK, the Sobs, Shannon‐Wiener, Inverse Simpson index, and Heip index of the plant community under HG decreased significantly by 23.78%, 37.97%, 47.43%, and 41.51%, respectively (*p* < 0.05). Simultaneously, the diversity indices of soil nematodes under HG also decreased significantly, being 22.2%, 40.3%, 50.9%, and 47.1% lower than those of CK, respectively. Linear and non‐linear correlation analyses demonstrated a significant positive correlation between plant diversity and nematode diversity, indicating a synergistic relationship between plant communities and soil nematode communities. Pearson correlation analysis revealed that *Cleistogenes songorica* (Roshev.) Ohwi and *Stipa breviflora* Griseb. were significantly positively correlated with the herbivorous nematode genus *Paratylenchus* (*p* < 0.05), while *Convolvulus ammannii* Desr. was significantly positively correlated with the genus *Acrobeloides* (*p* < 0.05). These results indicated that certain specific plant species exert significant regulatory effects on specific soil nematode species. Overall, heavy grazing is detrimental to the sustainable development of grasslands. Therefore, in light of previous research in this field, maintaining grazing intensity below the level of heavy grazing (LG or MG) is the most appropriate grassland management strategy.

## Graphical abstract

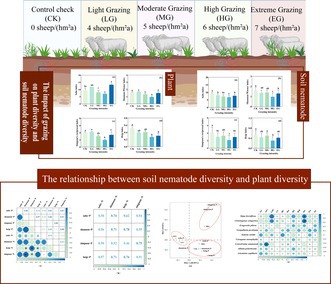

## 1 Introduction

Grasslands are the most widespread ecosystem in arid and semi‐arid regions, covering approximately 40% of the Earth's terrestrial surface (Zhao et al. 2020; Le Provost et al. 2023). In Inner Mongolia, the grasslands are a crucial component of the ecological structure, serving as a natural barrier that protects the northern part of China against desertification and climate change, playing an essential role in maintaining ecological balance (Reynolds et al. 2007). These grasslands are also vital for livestock production, as they provide forage for animals, contributing significantly to the PRC animal husbandry industry. Additionally, grasslands in Inner Mongolia offer a range of ecological services, including windbreaks, sand fixation, water and soil conservation, climate regulation, and biodiversity protection (White et al. 2000; Wang et al. 2017). However, the impact of sheep grazing, particularly in desert steppe ecosystems, has raised concerns regarding the sustainability of these grassland resources. Desert steppes, as one of the most arid ecosystems in Inner Mongolia, are highly sensitive to changes in grazing intensity (Council 1992; Wu et al. 2015). Due to their lower primary productivity and simpler community structure, these ecosystems are more prone to degradation under heavy grazing pressure (Li et al. 2008; Mcneely 2003). Grazing in desert steppes has been reported to result in changes in plant species composition, reduced plant biomass, and altered soil physical and chemical properties, leading to negative ecological impacts. The relatively poor stability and biodiversity of desert steppe further exacerbate its vulnerability to grazing (Deng et al. 2014).

Previous studies, assessing the impact of grazing in grasslands, have primarily focused on vegetation, soil properties, and ecosystem functions. The results have generally indicated that grazing affects plant diversity, vegetation cover, and soil quality, while also altering plant–soil interactions (Li et al. 2008; Mcneely 2003; Deng et al. 2014; Pei et al. 2008). Grazing‐induced changes in plant diversity have been shown to affect soil microbial communities, nutrient cycling, and organic matter decomposition. Despite these insights, the effects of grazing on soil nematodes in desert steppe ecosystems remain poorly understood. Nematodes, as abundant soil organisms that play key roles in nutrient cycling and organic matter decomposition, are considered effective bioindicators of soil health (Griffiths et al. 2001; Wang et al. 2015). Although grazing impacts on nematodes have been well‐documented in meadows and semi‐arid grasslands, little research has been conducted on their response in desert steppe ecosystems. Soil nematode communities reflect changes in soil structure, nutrient availability, and the impacts of grazing on ecosystem functions, making them a crucial component of soil biodiversity (Yeates 2003; Bongers and Bongers 1998). Studies from other regions have shown that grazing can reduce nematode diversity, affect functional groups, and alter community structure. However, the findings are inconsistent, with some studies suggesting no significant impact of grazing on nematode communities (Hu et al. 2015; Pan et al. 2022). This disparity may be due to the different environmental conditions, grazing intensities, and grassland types. In desert steppe ecosystems, which are characterized by low primary productivity and harsh environmental conditions, the effects of grazing on soil nematode communities may differ from those observed in other grassland types. Additionally, it is unclear whether the changes in soil nematode communities due to grazing are directly related to shifts in plant communities. Plants are key drivers of soil nematode populations, as they provide organic matter and create microhabitats for nematodes (Bardgett et al. 1997; Van Der Putten and Stoel 1998). Moreover, changes in plant diversity and biomass due to grazing disturbance can influence the abundance and composition of nematode communities. Previous studies have suggested that plant diversity and community structure affect nematode functional groups, such as herbivores and decomposers (Porazinska et al. 2003), but this relationship has not been well‐explored in desert steppe ecosystems.

Given the importance of both grazing management and soil health in desert steppe ecosystems, this study aims to investigate the effects of different grazing intensities on soil nematode biodiversity and explore the relationships between plant and nematode in desert steppes. By examining these interactions, the study seeks to provide a better understanding of the ecological processes in desert steppes under grazing disturbance and offers practical insights for sustainable grassland management.

## 2 Materials and Methods

### 2.1 Site Description and Experimental Design

**FIGURE 1:**
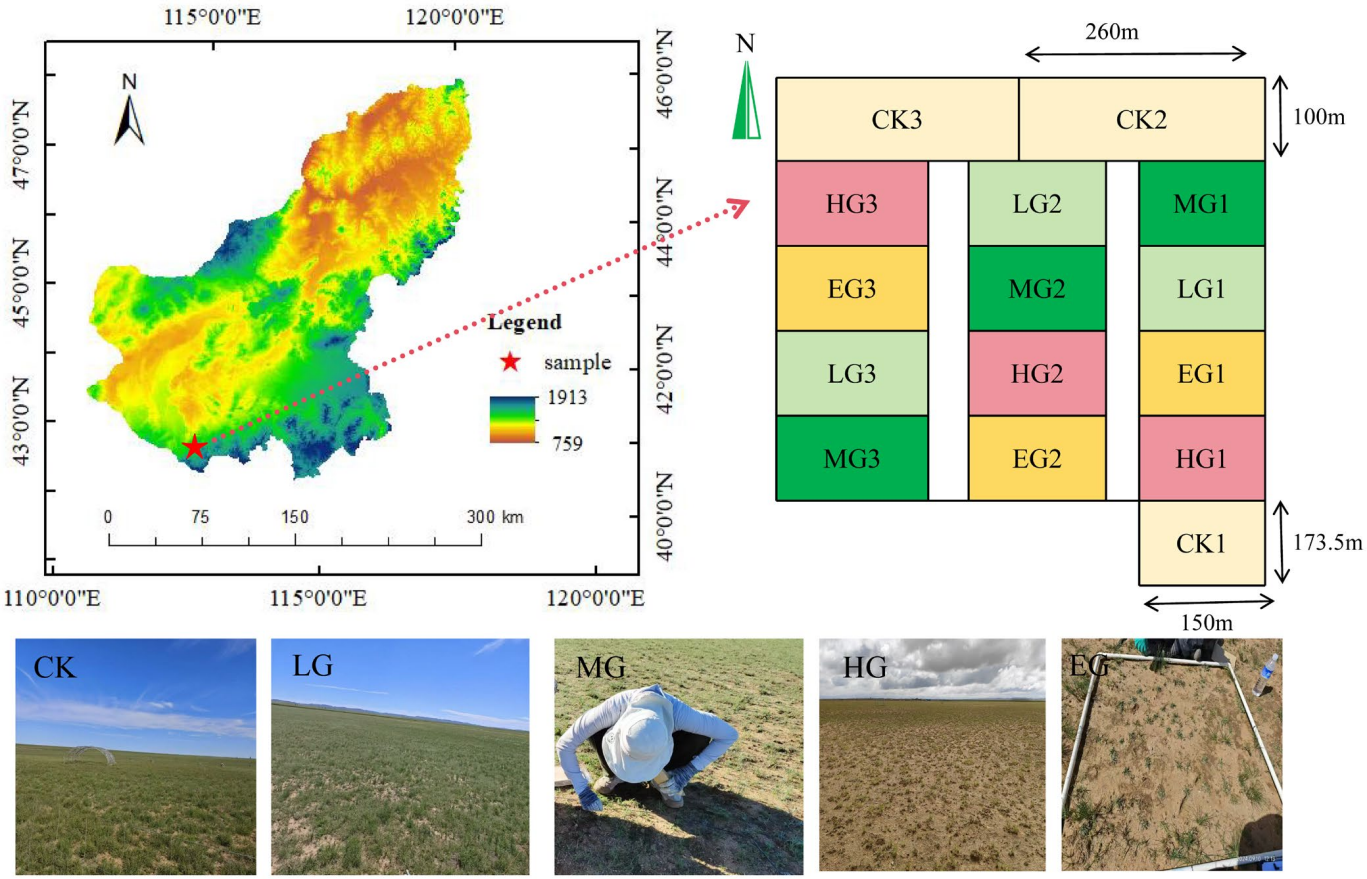
Distribution map of the experimental site. Grazing intensity treatments: Control (CK), Light grazing (LG), Moderate grazing (MG), Heavy grazing (HG), Extremely grazing (EG) represent 0, 1.54, 1.92, 2.31, and 2.69 sheep·hm^−2^a^−1^.

The study area is a desert steppe located at Hardenhushu Gacha in Inner Mongolia, China (112°70′26.92″ E, 42°20′69.2″ N; 1000–2000 m asl) (Figure 1). This region has a mid‐temperate semi‐arid continental climate, characterized by large temperature differences across seasons with dry, windy spring and hot, rainy summer. The summer lasts about 90 days, while the winter lasts about 120 days. This area has more than 270 frost‐free days and 3231.8 h of sunshine per year. During the period from 2020 to 2023, the average temperature was 5.97°C, the average precipitation was 180 mm, and the average evaporation was 2384 mm. The soil is characterized as a light chestnut calcic soil (Chinese Soil System Classification, CSSC) with a thickness of approximately 30 cm and a pH of 7.12.

The experimental area is located in the *Stipa breviflora* steppe, which is distributed in a strip along the southern part of the Inner Mongolia Plateau Desert Steppe Subzone. *Stipa breviflora* Griseb. serves as the constructive species in the community, playing a leading role and exerting a significant control over the structure and function of the plant community. It is a very palatable forage in early spring and late autumn, but its palatability is lower during the flowering and maturation stages, and it is highly tolerant to grazing. *Cleistogenes songorica* (Roshev.) Ohwi. and *Allium polyrhizum* Turcz. are also dominant species with a high grazing tolerance and palatability. Additionally, 13 subordinate plant species were found in this site (Table 1). Preferred forage with a relatively high intake ratio in the sheep diet mainly consists of annual and biennial forbs, as well as some perennial forbs (Zhang et al. 2018; He et al. 2022).

**TABLE 1:** Plant species composition, sheep palatability, and relative biomass (%) of the desert steppe community in the Inner Mongolia experimental site.

	Species	Functional groups	Palatability of plants	Grazing intensity				
				CK	LG	MG	HG	EG
Dominant	*Stipa breviflora* Griseb.	Perennial grasses	Moderate	13.293	17.253	14.657	7.915	8.301
Species	*Allium polyrhizum* Turcz. ex Regel	Perennial rhizome grasses	High	17.339	3.558	0.907	—	1.469
	*Cleistogenes songorica* (Roshev.) Ohwi	Perennial grasses	High	11.819	31.091	34.126	25.788	12.349
Subordinate	*Neopallasia pectinata* (Pall.) Poljakov	Annuals and biennials	Moderate	2.844	2.816	13.304	24.917	26.025
Species	*Convolvulus ammannii* Desr.	Perennial rhizome grasses	High	5.496	2.916	6.306	13.124	12.409
	*Eragrostis pilosa* (L.) P. Beauv.	Perennial rhizome grasses	High	5.996	3.946	14.289	4.171	7.044
	*Setaria viridis* (L.) P. Beauv.	Annuals and biennials	High	2.311	4.503	1.034	1.965	3.751
	*Caragana stenophylla* Pojark.	Shrubs and semi‐shrubs	Moderate	0.089	0.976	0.428	1.690	0.836
	*Kochia prostrata* (L.) C. Schrad.	Shrubs and semi‐shrubs	High	6.050	0.145	0.714	0.149	0.079
	*Astragalus galactites* Pall.	Perennial rhizome grasses	Moderate	0.106	—	—	0.945	0.288
	*Asparagus cochinchinensis* (Lour.) Merr.	Perennial rhizome grasses	Moderate	1.541	0.307	—	—	—
	*Iris tectorum* Maxim.	Perennial rhizome grasses	High	—	0.012	—	—	—
	*Lipschitzia divaricata* (Turcz.) Zaika, Sukhor. & N. Kilian	Perennial rhizome grasses	Moderate	0.007	—	—	0.079	0.119
	*Allium mongolicum* Regel	Perennial rhizome grasses	High	0.004	—	—	—	—
	*Allium tenuissimum* L.	Perennial rhizome grasses	High	0.007	—	0.097	—	—
	*Tribulus terrestris* L.	Annuals and biennials	High	—	—	—	—	0.068
	*Cleistogenes squarrosa* (Trin.) Keng	Perennial grasses	High	—	0.244	—	—	—

### 2.2 Experimental Design

A grazing experiment was set up with a complete randomized block design performed with five treatments and three replicates, making a total of 15 plots (2.1 ha per plot). Treatments included no grazing as a control check (CK) with 0 sheep·hm^−2^a^−1^, (throughout this paper, “a” is used to denote “one year”), light grazing (LG) at 1.54 sheep·hm^−2^a^−1^ (i.e., 4 sheep in the plot), moderate grazing (MG) at 1.92 sheep·hm^−2^a^−1^ (i.e., 5 sheep in the plot), heavy grazing (HG) at 2.31 sheep·hm^−2^a^−1^ (i.e., 6 sheep in the plot), and extreme grazing (EG) at 2.69 sheep·hm^−2^a^−1^ (i.e., 7 sheep in the plot) (Figure 1).

Grazing occurred from June to October what years: 2020–2023, between 6 a.m. and 6 p.m., with sheep left ungrazed at night and not given additional feed. Two‐year‐old sheep (55–60 kg) were selected for the grazing experiment and replaced annually.

Grazing intensity was calculated using the following equation:

$$\text{Grazing intensity}\;\left(\text{sheep}\cdot {\mathrm{hm}}^{-2}a^{-1}\right)=\frac{\text{number of livestock units}}{\text{sample plot area}\;\left({\mathrm{hm}}^{2}\right)}$$

*Note:* In this formula, “a” denotes “one year”.

### 2.3 Sampling

Plant and soil samples were collected in September 2022. For each grazing intensity, three replicate plots were established, totaling 15 plots. The plant community was surveyed in five randomly selected 1 m × 1 m quadrats within each plot. Plant species were morphologically identified by observing key plant features, such as leaf shape, stem texture, and floral structure. This process was supported by referring to taxonomic illustrations and literature for species identification. On this basis, for each quadrat, the number of plant species and the abundance of each species were recorded, and the plants were clipped at ground level and then placed into sample bags by species. The collected plant material was then dried in an oven at 65°C for 48 h and weighed by species to determine the biomass. Table 1 provides the basic information on plants. Simultaneously, one of the five quadrats was selected for the extraction of soil nematodes. A soil auger with a diameter of 7 cm was used to collect soil samples from a depth of 0–10 cm. The collected samples were promptly placed into sterile cryovial tubes for preservation and then sent to Shanghai Majorbio Bio‐Pharm Technology Co. Ltd. for sequencing to analyze the nematode community. Therefore, there were a total of 15 samples for nematode analysis, with three replicates corresponding to each grazing intensity. Table 2 provides the basic information on soil nematodes, respectively.

**TABLE 2:** Nematode community composition and relative abundance under different grazing disturbance intensities in the Inner Mongolia experimental site.

Trophic groups	Genus	*c*‐*p*	Abbreviation	Grazing intensity				
				CK	LG	MG	HG	EG
Bacteriovorus nematodes	*Acrobeles*	2	*Acr*	11.36	6.49	1.88	1.82	20
	*Acrobeloides*	2	*Acrs*	22.97	8.05	12.21	25.23	17.21
Phytophagous nematodes	*Paratylenchus*	2	*Par*	20.51	37.12	37.59	21.01	17.17
	*Heterodorus*	4	*Het*	33.86	26.75	34.76	25.06	—
	*Criconemoides*	4	*Cri*	3.22	6.07	—	9.68	—
	*Pratylenchoides*	4	*Pra*	1.47	1.61	0.17	—	20.4
	*Hoplolaimus*	4	*Hop*	0.79	1.28	—	8.57	0.53
	*Gracilacus*	4	*Gra*	—	—	—	—	2.13
	*Longidorus*	4	*Lon*	—	0.035	0.087	—	—
	*Rotylenchus*	4	*Rot*	—	0.45	—	—	—
	*Amplimerlinius*	2	*Amp*	—	0.21	—	—	0.66
	*Hoplotylus*	4	*Hops*	—	0.31	—	—	2.58
	*Malenchus*	2	*Mal*	—	0.28	—	—	—
	*Merlinius*	2	*Mer*	—	—	0.044	—	0.12
	*Miculenchus*	2	*Mic*	—	—	0.44	—	1.80
	*Pseudhalenchus*	2	*Pse*	—	1.04	—	—	—
	*Quinisulcius*	2	*Qui*	0.105	—	—	—	0.04
	*Schistonchus*	2	*Sch*	0.68	—	—	2.64	0.37
Fungivorus nematodes	*Aphelenchoides*	2	*Aph*	1.41	—	2.93	0.06	0.082
	*Ditylenchus*	2	*Dit*	0.21	1.39	0.61	—	2.95
	*Filenchus*	2	*Fil*	—	—	—	—	0.37
	*Aprutides*	2	*Apr*	—	1.01	—	—	—
Omnivore‐predatory	*Allodorylaimus*	4	*All*	0.31	—	—	—	—
	*Campydora*	4	*Cam*	0.11	7.15	3.53	—	—
	*Dorylaimellus*	5	*Dor*	0.84	0.38	—	5.93	0.29
	*Aporcella*	5	*Apo*	—	0.096	—	—	—
	*Pararhyssocolpus*	2	*Pars*	0.157	—	—	—	—
	*Robustodorus*	4	*Rob*	—	0.035	0.087	—	0.82

Total nematode genomic DNA was extracted from soil samples using the Fast DNA Spin Kit for Soil (MP Biomedicals, US) according to the manufacturer's instructions. The quality and concentration of DNA were determined by 1.0% agarose gel electrophoresis and a NanoDrop2000 spectrophotometer (Thermo Scientific, United States). The nematodes 18S rRNA gene was amplified with primer pairs NF1F (5′‐AGTACCGGTTCTATTCTAGAGGA‐3′) and 18Sr2bR (5′‐TTACACCTTGGTGTGAATGG‐3′) by a T100 Thermal Cycler PCR thermocycler (BIO‐RAD, USA) (Mago and Salzberg 2011). The PCR product that was extracted from 2% agarose gel was purified using the PCR Clean‐Up Kit (YuHua, Shanghai, China) according to the manufacturer's instructions and quantified using Qubit 4.0 (Thermo Fisher Scientific, USA). The purified PCR products were used for library construction with the NEXTFLEX Rapid DNA‐Seq Kit. Paired‐end sequencing was performed on the Illumina PE300 platform (Illumina, San Diego, USA) by Majorbio Bio‐Pharm Technology Co. Ltd. (Shanghai, China). Raw FASTQ files were de‐multiplexed using an in‐house Perl script, and then quality‐filtered by Fastp version 0.19.6 (Mago and Salzberg 2011). Also, make sure to avoid repetition (e.g., merging with FLASH) (Edgar 2013). After sequencing, 300‐bp paired‐end reads were generated. The raw data have been uploaded to the NCBI SRA database (Accession Number: PRJNA1269027). The quality control of raw paired‐end sequences was performed using Fastp (version 0.19.6) (https://github.com/OpenGene/fastp). Subsequently, FLASH (version 1.2.11) (Mago and Salzberg 2011) (http://www.cbcb.umd.edu/software/flash) was utilized to merge the paired‐end sequences. UPARSE (version 7.1) (Edgar 2013) (http://drive5.com/uparse/) was then employed to cluster the quality‐controlled and merged sequences into operational taxonomic units (OTUs) at a similarity threshold of 97%, while simultaneously removing chimeric sequences. To minimize the impact of sequencing depth on subsequent α diversity analyses, the sequence count for each sample was normalized to 20,000 (normalization is recommended). After normalization, the average Good's coverage for each sample remained at 99.09%. The RDP classifier (version 2.11) (Wang et al. 2007) (http://rdp.cme.msu.edu/) was used to annotate the OTUs against the Protist PR2 v5.0.1 database, with a confidence threshold of 70%. The community composition of each sample was then analyzed at different taxonomic levels.

### 2.4 Sampling Measurements

The ecological diversity indices of plant and nematode communities were calculated as follows:

(1) Sobs index:

Sobs=number of species observed

(2) Inverse Simpson index:

$$1/\lambda =1/\left(1-\sum pi^{2}\right)$$

$$\lambda =1-\sum pi^{2}$$

Note: *λ*: Simpson index.

In the subsequent research on the correlation between plant and nematode diversity, we used the Simpson index.

(3) Shannon‐Wiener index:

$$H=-\sum \mathrm{pi}\left(\ln\mathrm{pi}\right)$$

(4) Heip index:

$$E_{h}=\left[\exp\left(-\sum \mathrm{pi}\;\ln\mathrm{pi}\right)-1\right]/\left(s-1\right)$$

where *pi* denotes the proportion of the density of plant species/the proportion of the OTU of soil nematode species, *i* is the total density in the plant community/number of soil nematode species sequences contained in the OTU, and *s* is the number of plant species/number of all soil nematode species sequences. An OTU similarity level = 97% (0.97) was used for index evaluation.

### 2.5 Statistical Analysis

The α diversity of soil nematodes and plant communities was calculated using the Mothur version 1.30.2 platform, provided by Shanghai Majorbio Bio‐Pharm Technology Co. Ltd. (Edgar 2013; Wang et al. 2007; Schloss et al. 2009).

The effects of grazing intensity on ecological indices of soil nematodes and plants were evaluated using Duncan's multiple range tests with one‐way ANOVA (*p < 0.05*). Pearson's correlation analysis (linear correlation analysis) and gray relational analysis (nonlinear correlation analysis) were used to examine the relationships between plant and soil nematode α diversities. Correspondence analysis (CA) was conducted to explore the relationships between the community structure of soil nematodes, plant community structure, and grazing intensity. In the CA, the α diversity of soil nematodes at the genus level was the response variable, while plant diversity and grazing intensity served as the explanatory variables. All statistical analyses were performed using SPSS Version 18.0 (IBM Corporation, Armonk, NY, USA), and figures were generated using R 4.2.2 (Wang et al. 2007; Schloss et al. 2009; Rahman et al. 2023).

## 3 Results

### 3.1 Response of Plant and Soil Nematode α Diversity to Grazing Intensity

**FIGURE 2:**
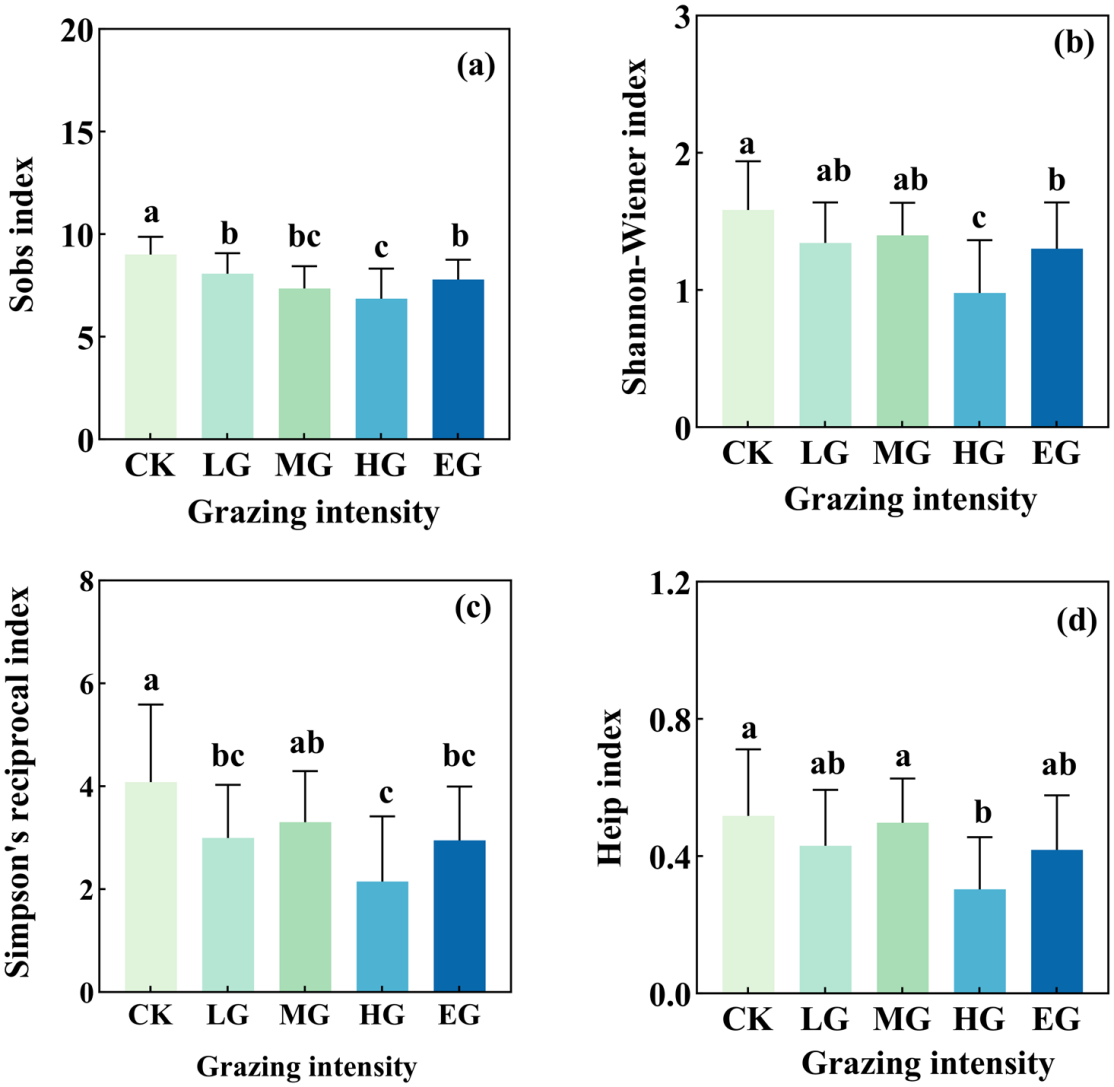
Plant species diversity under different grazing intensities. Barplots showing means for Sobs (a), Shannon‐Wiener (b), Simpson (c), Heip (d) indexes under different grazing intensities. Significant differences (*p* < 0.05) among grazing levels are indicated by different lowercase letters. Grazing intensity levels: As shown in Figure 1. Error bars represent the standard error.

**FIGURE 3:**
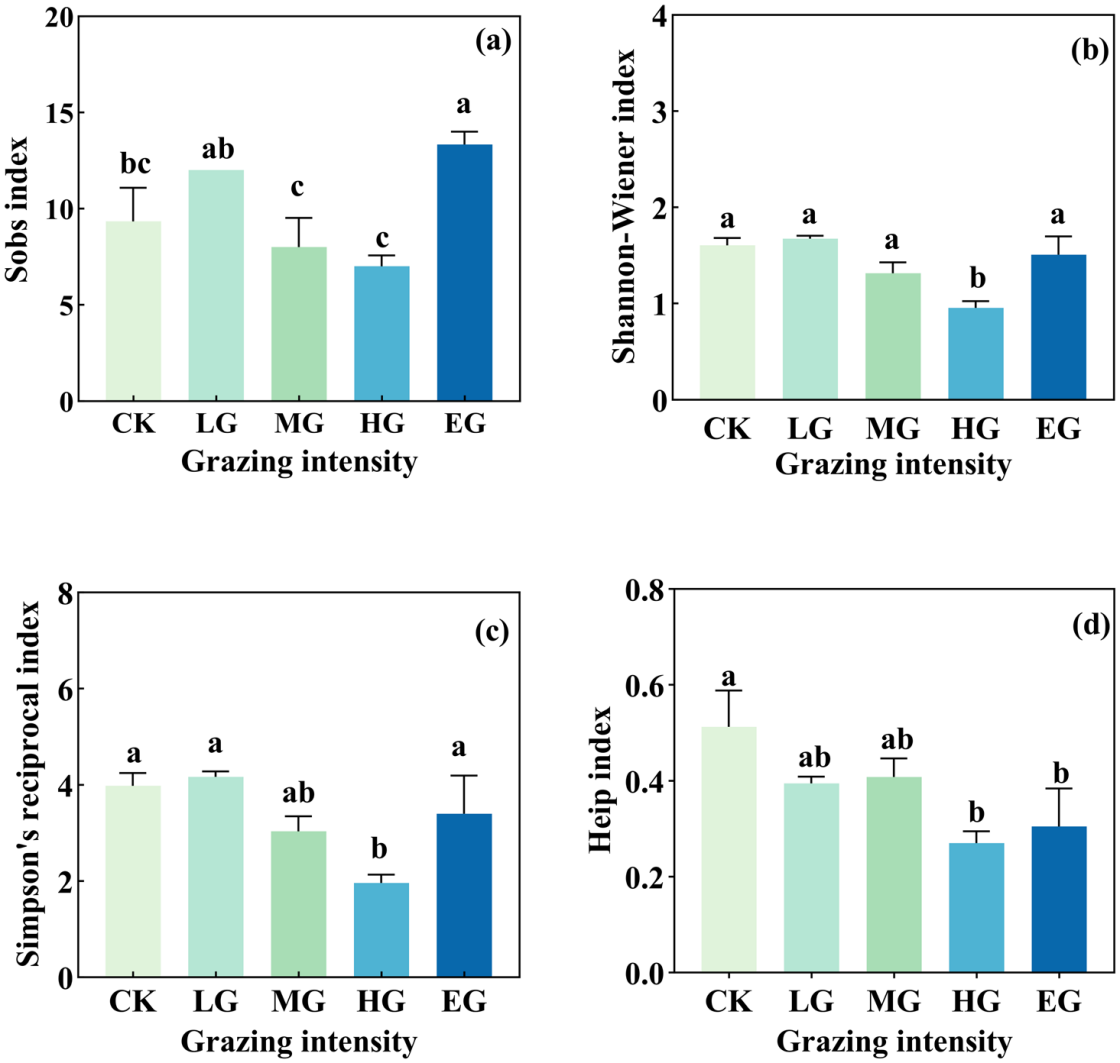
Nematode species diversity under different grazing intensities. Barplots showing Sobs (a), Shannon‐Wiener (b), Simpson (c), and Heip (d) indexes under different grazing intensities (mean ± SE). Significant differences (*p* < 0.05) among grazing treatments are indicated by different lowercase letters. For grazing intensity treatments: Grazing intensity treatments: See in Figure 1.

With increasing grazing intensity, the α diversity of plants and soil nematodes both showed a trend of initially decreasing and then increasing. Heavy grazing (HG) had a significant negative impact on the α diversity indices (including Sobs, Shannon‐Wiener, Simpson's reciprocal, and Heip's index) of both plants and soil nematodes (*p* < 0.05). Compared with the CK, the Sobs (Figure 2a), Shannon‐Wiener (Figure 2b), Inverse Simpson (Figure 2c) and Heip index (Figure 2d) of plant under HG decreased by 23.78%, 37.97%, 47.43% and 41.51%, respectively (*p* < 0.05). Compared with the EG, the Sobs and Shannon‐Wiener index of plant under HG decreased by 11.94% and 24.7% respectively (*p* < 0.05). The soil nematode α diversity, including the Sobs (Figure 3a), Shannon‐Wiener (Figure 3b), Inverse Simpson (Figure 3c), and Heip index (Figure 3d), were 22.22%, 40.3%, 50.9% and 47.1% lower, respectively, under HG than under CK (*p* < 0.05). Compared with EG, the Sobs, Shannon‐Wiener and Inverse Simpson index decreased by 46.15%, 36.42% and 42.35%, respectively, under HG (*p* < 0.05).

### 3.2 Correlation of α Diversity Between Plant and Soil Nematode Communities

**FIGURE 4:**
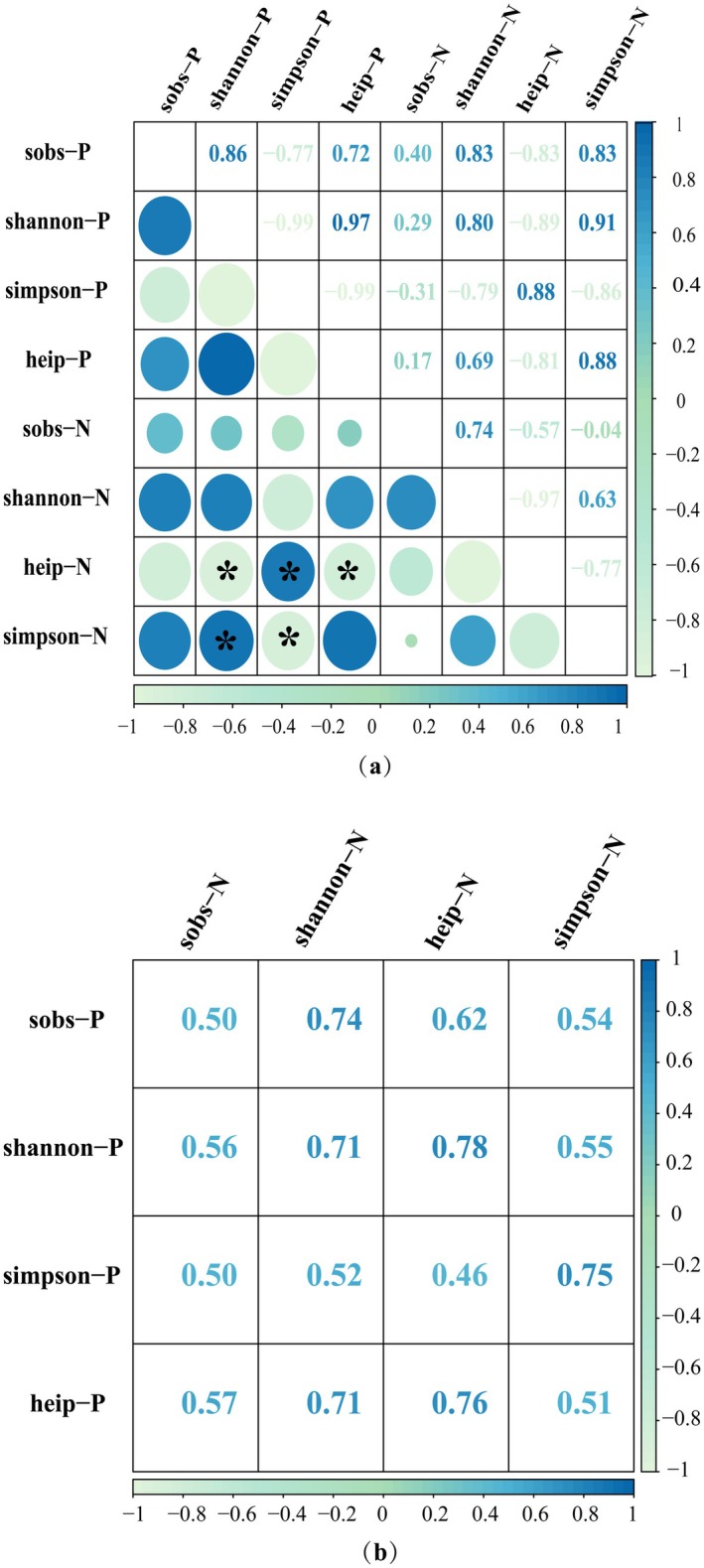
Pearson's correlation analysis (a) and gray relational analysis (b) based on the α‐diversity of soil nematode and plant communities. N and P represent soil nematode and plant communities, respectively. Significance indicated as **p < 0.01, and *p < 0.05. Color intensity indicates the strength of correlation, ranging from weak to strong: Light blue signifies weak correlation (correlation coefficient near 0), medium blue indicates moderate correlation, and the darkest blue denotes strong correlation (correlation coefficient near ±1).

Linear and nonlinear correlation analyses revealed consistent patterns in the relative values of plant and nematode α diversity. However, linear correlation indicated a negative relationship between the α diversity of plant and soil nematode communities (Figure 4a). Nonlinear correlation analysis highlighted the degree of correlation, though it did not specify the direction (Figure 4b). The Simpson index of plant communities showed a significant correlation with the Simpson index of soil nematode communities, suggesting that dominant species in plant communities correspond to dominant species in soil nematode communities. The Shannon‐Wiener index, Heip index, and Sobs index of plant communities were closely related to the Shannon‐Wiener and Heip indices of soil nematodes. Furthermore, changes in the Sobs index of soil nematodes were significantly correlated with the species diversity index of plant communities.

### 3.3 Correspondence Analysis Between the α Diversity of Plant and Soil Nematode Communities in Relation to Grazing Intensity

**FIGURE 5:**
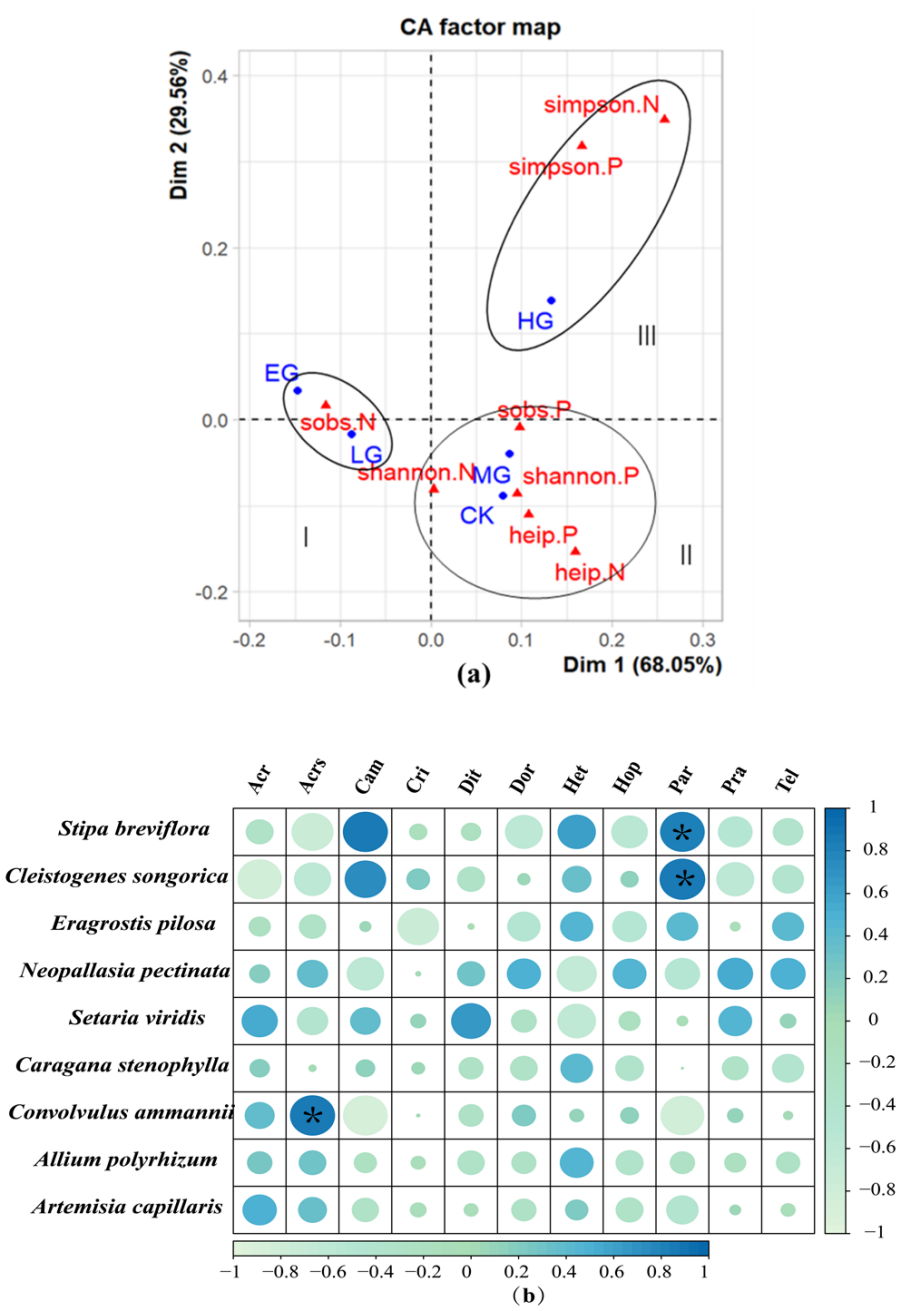
Correspondence analysis based on the α‐diversities of soil nematode and plant communities, and grazing intensity (a). Pearson's correlation analysis based on nematode community genera and plant densities (b). For grazing intensities see Figure 1. The full names of the nematode genera are shown in Table 2. Significance indicated as **p < 0.01; and *p < 0.05.

The correspondence analysis of α diversity in plant and soil nematode communities under various grazing intensities showed 97% accuracy in representing the original data, suggesting that two first factors can effectively replace the original data. The correspondence analysis factor map (Figure 5a) revealed three distinct regions. The first region (labeled I) indicated that light and extremely heavy grazing increased soil nematode species richness. The second region (labeled II) showed that control and moderate grazing enhanced the Shannon‐Wiener diversity and Heip evenness indices of both plant and soil nematode communities, as well as the Sobs richness index in plant communities. The third region (labeled III) indicated that heavy grazing increased the Simpson dominance index for both plant and soil nematode communities. Furthermore, Pearson's correlation analysis (Figure 5b) revealed significant correlations of *C. songorica* and *S. breviflora* with *Paratylenchus*, and between *C. ammannii* and *Acrobeloides*.

## 4 Discussion

### 4.1 The Impact of Grazing Intensity on Plant α Diversity

With increasing grazing intensity, the plant α diversity showed a trend of first decreasing and then increasing. HG significantly reduced the α diversity of plant communities (as indicated by Sobs, Shannon, Simpson's reciprocal, and Heip index diversity indices). Our results were consistent with previous research conducted in Siziwang Banner, Inner Mongolia (Zhang et al. 2018), as well as with findings that grazing reduced plant richness by 3.7% and evenness by 15.1% (He et al. 2022). Furthermore, our study partially supported a theoretical model linking grassland plant diversity responses to grazing intensity along moisture gradients and grazing evolutionary history (Liu et al. 2017). One possible explanation is that sheep preferentially graze on highly palatable and nutritious species such as *S. breviflora* and *C. songorica*, leading to a reduction in the overall community cover. This decrease in vegetation cover increases soil water evaporation, resulting in water stress that inhibits plant growth and reduces plant diversity (Wang et al. 2014). Another possible reason is that *S. breviflora* and *C. songorica* adopt avoidance strategies to grazing disturbance. Although their biomass decreases, they maintain their dominance under heavy grazing conditions (Yanling et al. 2016; Liu et al. 2016). Meanwhile, non‐dominant species experience different responses to grazing disturbance—*C. ammannii* and *N. pectinata* thrive due to reduced competition and poor palatability (Feng 2024), while others suffer biomass reductions or even local extinction. These changes led to an overall decrease in the α diversity of plant communities under HG. Interestingly, HG exerted a stronger impact on plant communities than EG. Additionally, EG showed similarities with other treatments, such as LG, MG, and even CK. It is possible that EG alters plant structure and species composition, creating opportunities for non‐dominant plant species to survive and establish themselves. As a result, the community underwent partial recovery and restructuring, leading to an increase in species richness and an overall enhancement in plant diversity.

### 4.2 Impact of Grazing Intensity on Soil Nematode α Diversity

The α diversity of soil nematodes exhibited a pattern of change similar to that of plant α diversity. HG significantly reduced the α diversity of soil nematodes. Our results aligned with a meta‐analysis showing that grazing significantly reduces the Shannon‐Wiener index (by 4.33%), the evenness index (by 9.22%), and species richness (by 5.35%) (Sun et al. 2024). Similarly, studies in semi‐arid grasslands found that grazing reduces the total abundance and diversity of soil nematodes (Pan et al. 2022; Bardgett et al. 1997; Van Der Putten and Stoel 1998; Porazinska et al. 2003; Zhang et al. 2018; He et al. 2022; Mago and Salzberg 2011; Edgar 2013; Wang et al. 2007, 2014, 2021; Schloss et al. 2009; Rahman et al. 2023; Liu et al. 2017, 2016; Yanling et al. 2016; Feng 2024; Sun et al. 2024). One possible explanation is that herbivorous nematodes constitute a major component of soil nematode diversity (Van Der Putten and Stoel 1998). These taxa obtain nutrients directly from plants or indirectly through the decomposition of plant litter (Grayston et al. 1998; Teshita et al. 2024). As grazing intensity increases, plant cover and biomass decline, leading to reduced litter input into the soil. This resource depletion has negative effects on plant diversity (Guo et al. 2021; Lv et al. 2024) and restricts root growth (Zhai et al. 2024). Since soil nematodes feed on plant roots and soil microbes, their reduction under HG could lead to a decline in soil organic‐matter inputs, and indirectly diminish nematodes that feed on them. This ultimately led to a significant reduction in soil nematode diversity (Pan et al. 2022). Interestingly, soil nematode α diversity under EG was significantly higher than that under HG. This may be due to the fact that under HG, soil nematodes may be affected by the loss of dominant plant roots, leading to a decline in diversity. However, when vegetation is largely destroyed in EG, and soil nematode habitat conditions become more uniform, leading to a more balanced species distribution and relatively higher diversity. Moreover, there may be more plant residues and organic matter in EG. These organic materials provide a richer food source for soil microbiovorous species, thereby promoting the diversity of soil nematodes.

### 4.3 Relationship Between Plant and Soil Nematode Communities Under Grazing

Plant communities serve as the primary resource base for soil biota, making plant composition and diversity closely linked to soil organisms (Bardgett and Wardle 2003; Veen et al. 2010). We simultaneously assessed plant and soil nematode responses to grazing disturbance, revealing significant interrelationships between these communities. Linear and nonlinear correlation analyses indicated a synergistic variation between plant and soil nematode communities, with a strong correlation between the Simpson dominance index of plant and soil nematode communities. This suggests that dominant species in plant communities correspond to specific dominant species in soil nematode communities. Further analysis showed that *C. songorica* and *S. breviflora* were significantly correlated with the herbivorous nematode genus *Paratylenchus* (*p < 0.05*), while *C. ammannii* was significantly correlated with the genus *Acrobeloides* (*p < 0.05*). These findings support previous studies showing that changes in dominant plant species can significantly affect soil nematode diversity (Nielsen et al. 2014). Furthermore, specific plant species may promote the abundance of particular plant‐feeding nematode species (Deyn et al. 2004). Soil nematodes interact with plants in multiple ways: some species directly feed on roots, while others rely on root exudates, decaying plant matter, and leaf litter (Wardle et al. 2004; Bardgett et al. 2001). Moreover, herbivorous nematodes often exhibit host specificity (Van Der Putten and Stoel 1998). Given the similar root exudate profiles of *C. songorica* and *S. breviflora* , *Paratylenchus* exhibits specificity toward both species. Meanwhile, *C. ammannii* root exudates significantly reshape soil microbial communities through low‐molecular‐weight organic acids, which alter nutrient forms and availability. This enhanced bacterial environment may specifically promote *Acrobeloides* abundance across its life stages (Gao 2017). These results highlight the intricate interactions between plant and soil nematode communities under grazing pressure, underscoring the importance of plant composition in shaping soil biodiversity.

## 5 Conclusion

Both diversities significantly declined under HG, indicating that HG has a significant negative impact on the soil ecosystem. Moreover, a significant and positive correlation was found between plant and soil nematode diversities, suggesting a close interaction between plant and soil nematode communities. Specifically, *C. songorica* and *S. breviflora* were significantly correlated with the herbivorous nematode genus *Paratylenchus* (*p* < 0.05), while *C. ammannii* was significantly correlated with the genus *Acrobeloides* (*p* < 0.05). These findings further demonstrate that specific plant species may have a significant regulatory effect on specific soil nematode species.

Overall, HG is highly detrimental to the sustainable development of grasslands. Therefore, we suggest that grazing intensity should be maintained at LG or MG levels. By integrating the relationship between plants and soil nematodes, our research findings provide significant practical guidance to improve the soil health of desert steppe to achieve sustainable grassland development.

## Author Contributions

**Rui Dong:** conceptualization (lead), data curation (lead), formal analysis (lead), investigation (lead), writing – original draft (lead). **Yanling Wu:** funding acquisition (equal), investigation (equal), supervision (equal), writing – review and editing (equal). **Shijie Lv:** data curation (equal), formal analysis (lead), investigation (equal), methodology (lead), supervision (equal), writing – review and editing (equal). **Changlin Xue:** project administration (equal), supervision (equal). **Wentao Wang:** visualization (equal). **Jie Yun:** supervision (equal).

## Conflicts of Interest

The authors declare no conflicts of interest.

## Supporting information


**Data S1:** ece372079‐sup‐0001‐DataS1.xlsx.

**Data S2:** ece372079‐sup‐0002‐DataS2.docx.

## Data Availability

The Data and code supporting the results of this study are saved in “Data” and “R‐code”.
