# Supplementary material for: Heavy Grazing Leads to Increased Dominance of Plant and Soil Nematode Communities
Source: Ecol Evol. 2025 Aug 31;15(9):e72079. doi: 10.1002/ece3.72079 (PMC12399570; doi:10.1002/ece3.72079)
Supplement: Supplementary file 2 — Data S2: ece372079‐sup‐0002‐DataS2.docx. [file ECE3-15-e72079-s001.docx]

**Plant Diversity ANOVA Code**/**Nematode Diversity ANOVA Code**

library(readxl)

library(ggplot2)

library(dplyr)

workbook <- ""

mydataframe <- read_xlsx(workbook,1)

plotdata <- mydataframe %>%

group_by(``)%>%

summarise(n=n(),mean=mean(``,na.rm=TRUE),se=sd(``)/sqrt(n))

plotdata

p <- ggplot(plotdata, aes(x=``, y=mean, fill=``)) +geom_bar(stat="identity") +scale_fill_manual(values = c("A" = "#E0F3DB", "B" = "#B5E6D4", "C" = "#A8DDB5","D"= "#4EB3D3")) +geom_errorbar(aes(ymin=mean-se, ymax=mean+se), width=0.2)+labs(x="", y=" ") + theme_bw()+theme(panel.grid = element_blank(),legend.position = "none",text = element_text(family = "serif", face = "bold"),axis.text = element_text(family = "serif", face = "bold"),legend.text = element_text(family = "serif"))+scale_y_continuous(name = "",breaks = seq(0,200,50),minor_breaks = seq(0,200,50),limits=c(0,200))+scale_x_discrete(name = "",limits=c("A","B","C","D"),labels=c("CK","LG","MG","HG"))

a <- p + annotate("text", x = 1, y = 190.1, label = "ab", size = 4, family = "serif")+annotate("text", x = 2, y = 185.1, label = "b", size = 4, family = "serif")+annotate("text", x = 4, y = 190.1, label = "ab", size = 4, family = "serif")+annotate("text", x = 3, y = 195.1, label = "a", size = 4,

**Person Correlation Analysis and Association Degree Analysis Code**

library(corrplot)

library(readxl)

library(ggplot2)

workbook <- ""

mydataframe <- read_xlsx(workbook,8)

mydataframe <- as.data.frame(mydataframe)

row.names(mydataframe) <- mydataframe[, 1]

df2<-mydataframe[,-1]

df2<-as.matrix(df2)

corrplot.mixed(df2, lower = 'circle', upper = 'circle')

col <- colorRampPalette(c("#A8DDB5","#0868AC"))(5)

par(family = "serif")

corrplot(df2, method = "number", type="lower",col = col, tl.col = "black", tl.cex = 0.7, tl.srt = 45, tl.pos = "l",add=T,addgrid.col = "black")

corrplot(df2, method = "number", type="upper",col = col, tl.col = "black", tl.cex = 0.7, tl.srt = 45, tl.pos = "lt",add=T,addgrid.col = "black")

rownames(df2) <- c("sobs-P", "shannon-P", "simpson-P", "heip-P", "sobs-N", "shannon-N", "heip-N", "simpson-N")

**Correspondence Analysis code**

library(readxl)

library(FactoMineR)

library(CA)

x=read_excel()

y=data.frame(x)

rownames(y)=y$cd

z=y[-1]

rea.ca=CA(z)
